# Implementation of an in‐house visual feedback system for motion management during radiation therapy

**DOI:** 10.1120/jacmp.v17i1.5817

**Published:** 2016-01-08

**Authors:** Vi Nhan V. Nguyen, David C. Ellerbusch, Ashley J. Cetnar, Joshua A. James, Brian Wang

**Affiliations:** ^1^ Department of Radiation Oncology University of Louisville School of Medicine Louisville KY; ^2^ Department of Radiation Oncology Mayo Clinic Rochester MN USA

**Keywords:** respiratory gating, in‐house, video goggles feedback, Varian RPM

## Abstract

In this Technical Note, we describe an in‐house video goggles feedback system assembled using several commercially available products. This goggle video feedback system is currently being used at University of Louisville and Mayo Clinic for both CT simulation and linac treatment delivery. The setup details, including specific recommendations, are provided, along with an alternative option for using the video goggles system.

PACS number: 07.07Hj

## I. INTRODUCTION

Respiratory gating and breath‐hold techniques are commonly used in radiation therapy to reduce the dose to the surrounding normal tissues and/or to limit the tumor's motion caused by the patient's breathing. Several studies have demonstrated that an external feedback coaching system can help patients maintain and reproduce breathing patterns during treatment, thus improving the delivery accuracy and efficiency.^1^, ^2^, ^3^, ^4^, ^5^, ^6^, ^7^, ^8^, ^9^, ^10^, ^11^ Common coaching systems typically involve the use of an audio‐ or video‐feedback signal, or a combination of both, to control and regulate patient's respiratory patterns. Haasbeek et al.^(9)^ reported an average increase on end‐inspiration lung volumes of 10.2% when comparing the simulation CTs created with and without an external audio coaching system, thus improving dosimetric sparing of normal tissue. Goossens et al.^(10)^ demonstrated that breathing patterns and tumor motion were controlled better and reproduced more easily with the aid of an audio and in‐house video goggles feedback system. The improvement in delivery efficiency was quantified by Linthout et al.,^(11)^ who showed that the average treatment delivery time of a gated stereotactic body radiation therapy (SBRT) treatment for a free‐breathing case could be reduced from 1.7 min to 1.4 min per 100 MU (standard deviation (SD)=0.6 min/100 MU) with the aid of a visual feedback system, and further reduced to 0.9 min per 100 MU (SD=0.2 min/100 MU) with an addition of an audio coaching system.

Besides the above‐mentioned investigations, other studies have also demonstrated the benefits of the video feedback system for motion management.^12^, ^13^, ^14^ The visual feedback systems from all these studies are built in‐house. Currently, what is missing in the literature are the details on how these systems are assembled. It is noted that an audio coaching system can be purchased from the vendors; however, a visual feedback system is not currently supplied or supported by any vendors or suppliers. In this study, we will describe the details of an in‐house video goggles feedback system assembled from several commercially available components. This video goggles feedback system is currently being used at two institutions on both the simulation computed tomography (CT) scanners and treatment linear accelerators (linacs) for all breath‐hold patients. The goal of this paper is to share our experiences with this system, provide details on how the system is set up, describe what equipment is needed, and provide the suggested user settings on each component. Also, readers should be aware that the system is not supported by any linac vendors at the time of publication.

## II. MATERIALS AND METHODS

**Figure 1:**
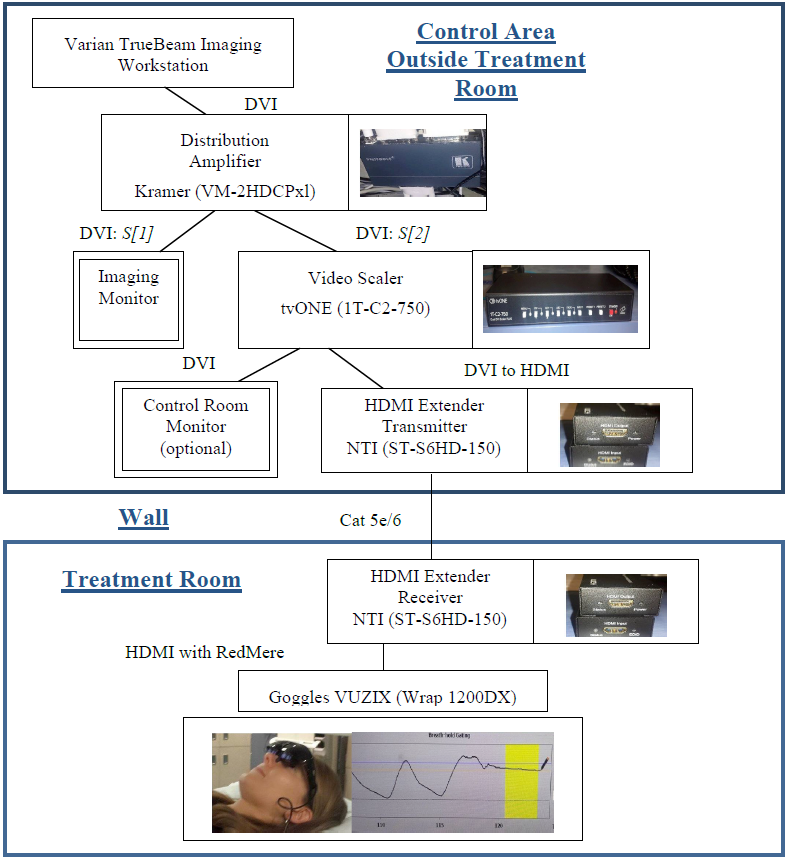
The connection diagram for the in‐house video goggles feedback system. The length of the cable is dependent on the design of the vault.

As depicted in Fig. 1, this video goggles system (Vuzix Wrap 1200DX goggles; Vuzix, West Henrietta, NY) works by splitting and amplifying the video output signal directly from the Varian Real‐time Position Management (RPM) workstation or TrueBeam Respiratory Motion Management (RMM) workstation (Varian Medical Systems, Palo Alto, CA) into two signals, using a distribution amplifier.

**Figure 2:**
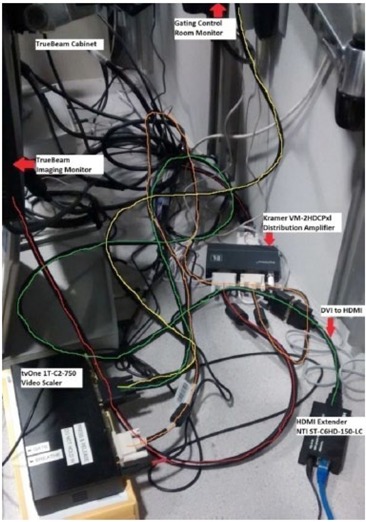
The wiring diagram for the in‐house video goggles feedback system with color‐coded cables in the console area outside of treatment room.

The first signal (S[1]) is reconnected back to the imaging monitor for normal workflow. The second signal (S[2]) is connected to the input of a video scaler to direct the signal to the goggles system and to an optional control room monitor. The video scaler is used to define the portion of the display the patient will see in the video goggles. The optional control room monitor is used to display the same video display that the patient is seeing on the video goggles. A dual DVI splitter should be purchased if the optional control room monitor would be used. The output signal S[2] from the video scaler is then connected to an HDMI extender transmitter via a DVI‐D to HDMI converter cable. Figure 2 shows a wiring diagram of the console area outside of the treatment room. S[2] is then sent from the HDMI extender transmitter to the HDMI extender receiver located inside the treatment room via a cat5e/6 cable.

Inside the treatment room, the HDMI extender receiver is permanently mounted on the wall near the conduit where the cat5e/6 cable is located. It is recommended that the location of the HDMI extender receiver be away from the primary beam to minimize the damage from radiation to the electronics. It is also recommended that the location of the HDMI extender receiver can be easily accessed by the therapists. An HDMI cable is used to connect from the output of the HDMI receiver to the video goggles.

## III. RESULTS & RECOMMENDATIONS

At University of Louisville, the system was just recently implemented for treatments of six breath‐hold gated patients on a Varian TrueBeam over an eight month period. At Mayo Clinic, the system was used to treat more than 100 breath‐hold gated patients on six Varian TrueBeam linacs and has been operational for two years. Our experience with the system showed that the patients tolerated it very well. The system has also been implemented for CT simulation of breath‐hold gated patients using the Varian RPM system and can be implemented on linacs other than the Varian TrueBeam, as well. The average time to prepare the goggles for treatment or simulation is approximately 1 minute. While we provide a few recommendations on how to test the functionality of the system, our main focus is to present the details on how to implement this in‐house system into clinical practice.

## RECOMMENDATIONS

### A. The video goggles

The Vuzix Wrap 1200DX goggles used in our video goggles feedback system come with a rechargeable Li‐ion battery pack and a USB charging cable. We recommend that a USB connecter plug should be purchased separately so that power can be provided to the goggles in case the battery runs low while in use.

### B. The distribution amplifier

Occasionally, the imaging signal from the linac will turn off if it detects a different output connection than the monitor due to a difference in the extended display identification data (EDID). Therefore, it is recommended that users program the distribution amplifier to store the desired EDID. The amplifier has a built‐in EDID management system that reads and stores the EDID from the linac's imaging monitor. Readers can refer to the user manual for a more detailed instruction on how to program the EDID for the amplifier.

### C. The video scaler

The video signal can be scaled, cropped, and panned in real time to display only the relevant information on the monitor to the patient. The tvONE1T‐C2‐750 video scaler (tvONE, Margate, UK) has two preset slots that can be used to program and store the settings. The customized settings can be varied depending on the manufacture and software version of the linac and Varian RPM system at the CT. The preset settings described in Appendix A (Table A1) is recommended to use to set the patient's breathing waveform for TrueBeam (version 2.0) linac.

The video scaler must be set with the proper output resolution (1280×720 60Hz) to match the goggles' input resolution. It is highly recommended to have an external monitor available when first commissioning the video goggles feedback system if the optional control room monitor is not implemented. Once the output resolution is set, the settings can be stored in the preset slots in the video scaler. The signal can then be displayed in the goggles to adjust other customized settings such as zoom, crop, and pan.

The video scaler has the option to display a stored static image for the patient to see during treatment when it is not necessary for patients to view their waveform. The software can be downloaded from the tvONE support software website.^(15)^ A 9‐pin serial cable is needed along with the “CORIOtool Suite‐C2” software to upload the image. The uploaded image can be displayed during the kV‐kV or CBCT image matching process by pressing the fade button on the scaler. A black screen will be displayed if an image is not available.

### D. Recommended synchronization test

After the system is completely set up, it should be verified that the desired video screen of the patient's breathing waveform can be seen on the goggles' display. The goggles should be tested on the treatment couch with a volunteer prior to implementation. Several breathing patterns should be conducted and visually confirmed to check the waveform's amplitude and period, and to verify that the waveform on the goggle's display is correctly synchronized with the TrueBeam RMM workstation's monitor. The steps can be repeated at the Varian RPM system on the CT simulator to ensure the same level of synchronization can be achieved. Other quality assurance procedures such as signal reproducibility, image quality, and hardware and cable performance checks should be conducted to ensure the safety of the system. A review of the studies conducted by Jiang et al.^(12)^ and Galvin and Bednarz^(13)^ are recommended.

#### B.1 Alternative option

An alternative way is to use a goggles system with input from a tablet (if the readers don't want to change their current linac setup). For example, an Apple iPad operating in camera mode is placed on a stand in front of the monitor displaying the patient's respiratory waveform. The video signal from the iPad camera is converted from the 30‐pin to VGA so that it can be input to the VR2200 goggles system (Virtual Realities LLC, League City, TX). (This iPad video goggles system was used at University of Louisville prior to implementing the current in‐house goggle system.) The goggles system is powered by an AC adapter provided with the goggle system. The disadvantage of this system is the instability of the setup. The iPad is held on a stand that can be easily displaced. Because the zoom is provided manually on the touch screen, subtle movement or adjustment results in a large change viewed by the patient. The quality of the video viewed by the patient is dependent on the angle of the iPad to the monitor and the lighting of the room. Since the iPad needs to be set up at the beginning of every treatment, the cable connections are less stable compared to the current option.

## Supporting information

Supplementary Material

Supplementary Material

Supplementary Material

Supplementary Material

## Appendix

**Table A1:** The patient's breathing waveform and gating limits can be set to display on the video goggles. The settings for main menu and submenu to adjust the output, window, source, and transition options on the video scaler are shown. These presets are recommended for use in the video scaler, to display the patient's wave function in the video goggles.

*Menu Group Name*	*Submenu*	*Group Description*	*Customer User Settings*
*Adjust Outputs*		*Controls output parameters*	*Yes*
	Lock mode	The output resolution is “locked” or the same as the input resolution	[OFF]
	Output res.	The top line of the display shows the current output resolution selected	1280x720 60 Hz [46]
	HDCP	High‐bandwidth Digital Content Protection	[ON]
	Output type	The analog and digital outputs are controlled separately (setting for the analog output)	[RGBHV]
	Back Y/U/V	Sets the value of the fixed background color	[16][128][128]
*Adjust Windows*		*Controls characteristics of the scaled window*	*Yes*
	Window to adjust	To select which window you want to modify: A, B, or Z. Windows A & B can be scaled (Zoom, Shrink, Pan and Crop)	[A]
	Source	Sets the input source (DIV‐I 1 or DVI‐I 2) for the currently selected window. Sill Image Stores (SIS1 or SIS2) can also be a static source	[DVI‐I 1]
	Window enable	This quickly enables or disables the window being adjusted	[ON]
	Zoom level (%)	Sets the amount of picture magnification you wish to use for the window source. You are provided with the options to zoom the image from 100% to 1000% (10x zoom)	[290]
	H/V zoom pan (%)	Once the image has been ‘zoomed’, this control allows the image to be positioned within the window so that any portion can be seen, not just the middle	[66][76]
	Image freeze	Allows the image to be frozen or unfrozen	[OFF]
	H/V crop (%)	This allows the scaled image to be cropped at the top/bottoms edges, or at the sides	[4][15]
	Shrink level (%)	Determines the percentage of the monitors' total available screen space that the selected Window image occupies	[60%][OFF]
	H/V position (%)	Determines the position of the shrunken image on the monitor screen	[50][40]
	Aspect change	Change the output screen aspect ratio, to suit the final output display size	[Normal]
	Layer priority	Selects the order of the window layers	ABZ[1]
*Adjust Keyers*		*Controls the keying ability of the unit*	*No*
*Adjust Borders*		*Controls the window border functions*	*No*
	Source to adj.	Selects the input connection for which you want to make adjustment to	[DVI‐I 1]
	HDCP	High‐Bandwidth Digital Content Protection	[ON]
	Auto set status		[Inactive]
	Aspect correct	To correct the aspect ratio of the video source when converted into the final output resolution	[H‐fit]
*Adjust Transition*		*Controls input transitions*	*Yes*
	Transitions	Controls the type of transition desired: Cut, Fade, Wipe, or Push	[Fade]
	Transition time	Controls how long a transition from one input to another takes	[0.5]
*Adjust Buttons*		*Controls the functions of the buttons*	*No*
*System*		*Controls global system parameters for the unit*	*No*
