# Supplementary figures and images for: Implementation of an in‐house visual feedback system for motion management during radiation therapy
Source: J Appl Clin Med Phys. 2016 Jan 8;17(1):421–7. doi: 10.1120/jacmp.v17i1.5817 (PMC5690230; doi:10.1120/jacmp.v17i1.5817)

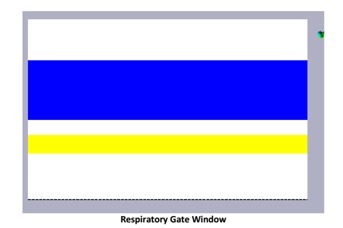

Supplement: Supplementary file 1 — Supplementary Material [file ACM2-17-421-s001.jpg]

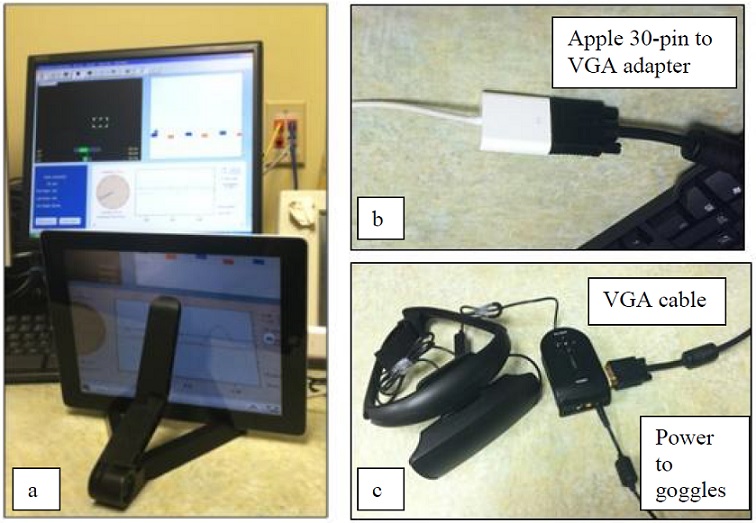

Supplement: Supplementary file 2 — Supplementary Material [file ACM2-17-421-s002.jpg]

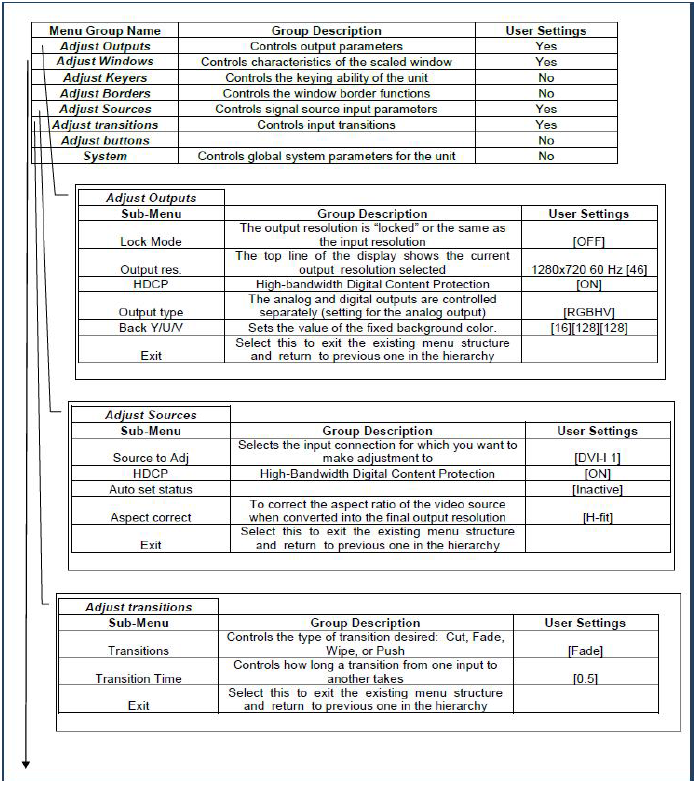

Supplement: Supplementary file 3 — Supplementary Material [file ACM2-17-421-s003.png]

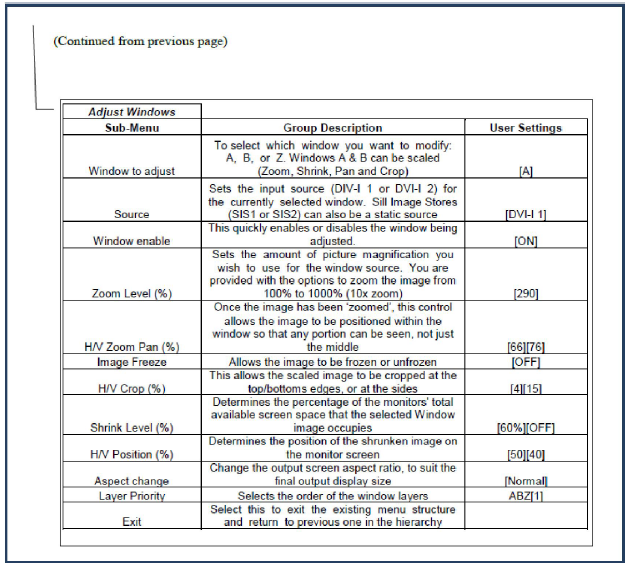

Supplement: Supplementary file 4 — Supplementary Material [file ACM2-17-421-s004.png]
